# The evolution of autotomy in leaf‐footed bugs

**DOI:** 10.1111/evo.13948

**Published:** 2020-04-08

**Authors:** Zachary Emberts, Colette M. St. Mary, Cody Coyotee Howard, Michael Forthman, Philip W. Bateman, Ummat Somjee, Wei Song Hwang, Daiqin Li, Rebecca T. Kimball, Christine W. Miller

**Affiliations:** ^1^ Department of Biology University of Florida Gainesville Florida 32611; ^2^ Florida Museum of Natural History University of Florida Gainesville Florida 32611; ^3^ Entomology and Nematology Department University of Florida Gainesville Florida 32611; ^4^ Behavioural Ecology Lab School of Molecular and Life Sciences Curtin University Perth WA 6845 Australia; ^5^ Smithsonian Tropical Research Institute Balboa Panama; ^6^ Lee Kong Chian Natural History Museum National University of Singapore Singapore 117377 Singapore; ^7^ Department of Biological Science National University of Singapore Singapore 117543 Singapore

**Keywords:** Autotomy, evolutionary ecology, evolutionary origins, latitudinal gradient, phylogenetic comparative methods, predator‐prey

## Abstract

Sacrificing body parts is one of many behaviors that animals use to escape predation. This trait, termed autotomy, is classically associated with lizards. However, several other taxa also autotomize, and this trait has independently evolved multiple times throughout Animalia. Despite having multiple origins and being an iconic antipredatory trait, much remains unknown about the evolution of autotomy. Here, we combine morphological, behavioral, and genomic data to investigate the evolution of autotomy within leaf‐footed bugs and allies (Insecta: Hemiptera: Coreidae + Alydidae). We found that the ancestor of leaf‐footed bugs autotomized and did so slowly; rapid autotomy (<2 min) then arose multiple times. The ancestor likely used slow autotomy to reduce the cost of injury or to escape nonpredatory entrapment but could not use autotomy to escape predation. This result suggests that autotomy to escape predation is a co‐opted benefit (i.e., exaptation), revealing one way that sacrificing a limb to escape predation may arise. In addition to identifying the origins of rapid autotomy, we also show that across species variation in the rates of autotomy can be explained by body size, distance from the equator, and enlargement of the autotomizable appendage.

Predation can impose strong selection. As a result, animals have evolved extraordinary defenses (i.e., antipredatory traits) that reduce their vulnerability, contributing to the morphological and behavioral diversity we see in organisms today (Caro 2005; Ruxton et al. 2018). One of the most extreme forms of antipredatory defense is autotomy, where individuals literally sacrifice part of their body while attempting to escape. A lizard dropping its tail in response to a predator attack is an iconic example (Arnold 1984; Bateman and Fleming 2009). However, autotomy also occurs in a diversity of other organisms: squid amputate their arms (Bush 2012), harvestmen release their legs (Guffey 1999), and salamanders drop their tails (Maiorana 1977). Despite having multiple origins (Emberts et al. 2019), fundamental questions about the evolution of autotomy remain unanswered. Most notably, how does sacrificing a limb to escape predation evolve, and what factors promote and constrain autotomy's evolution?

Autotomy is predominantly thought of as an antipredatory trait, but there are two additional survival benefits associated with autotomy: reducing the cost of injury (Emberts et al. 2017a) and escaping nonpredatory entrapment (Maginnis 2008). Several species have been observed autotomizing limbs that have been severely damaged (e.g., after intraspecific competition or a failed predation event), and this reduces mortality (Emberts et al. 2017a). Escape from nonpredatory entrapment (Maginnis 2008) may also be a widespread benefit, especially in the Ecdysozoa (i.e., arthropods, nematodes, and allies; Fleming et al. 2007; Hodgkin et al. 2014). Within this clade, nonpredatory entrapment frequently manifests itself as entrapment in a fouled molt, which an organism can escape through autotomy (Wood and Wood 1932).

Although escaping nonpredatory entrapment and reducing the cost of injury are rarely discussed, these benefits may be crucial to understanding the evolution of autotomy because these two benefits do not require individuals to autotomize quickly. In fact, autotomy can occur several minutes, and potentially even hours, after an incident occurs (i.e., entrapment and injury) and still be beneficial (Emberts et al. 2017a). On the other hand, individuals need to be able to drop their limbs quickly to use autotomy in an antipredatory capacity. As a result, it has been hypothesized that escaping predation is *not* the ancestral benefit of autotomy (McVean 1982). Instead, McVean (1982) hypothesized that sacrificing a limb to escape predation is a co‐opted benefit, and that autotomy to reduce the cost of injury is an ancestral state (i.e., benefits associated with autotomizing slowly are an intermediate step in the evolution of much more rapid autotomy). Alternatively, autotomy's evolution may be exclusively associated with dropping a limb quickly enough to escape predation (i.e., the fast latency hypothesis; Emberts et al. 2019). Note that the fast latency hypothesis states that a lineage transitions from being unable to autotomize to being able to autotomize quickly (i.e., there is no intermediate step with regards to the *rate* of autotomy). The first aim of this study is to investigate these two alternative hypotheses for the origins of rapid autotomy.

The second aim of our study is to investigate the ecological and morphological factors associated with autotomy's evolution. Three major factors that are thought to influence autotomy are (1) predation, (2) the costs associated with autotomizing, and (3) body size. Autotomy is not an effective strategy against all predator classes. For example, tail autotomy in some lizards does not defend against predation by falcons, but it is effectively used against teiids (i.e., a predatory lizard) and snakes (Medel et al. 1988). Similar patterns have also been found in crickets, where autotomy is more effective against mice than against skinks (Bateman and Fleming 2006a). As a result, the predatory selection acting on autotomy depends on the abundance of certain predator species. One broad‐scale metric that has been used to capture this predatory selection is predator diversity, which has been shown to positively correlate with the ease of tail autotomy in lizards (Cooper et al. 2004; Brock et al. 2015). Although predation is thought to promote autotomy, the cost of losing the autotomizable limb, even temporarily, potentially constrains it. This constraint is most discernible when a sexually selected trait is on an autotomizable appendage, as autotomy results in the costly loss of a trait that is important for reproductive success (discussed in Wasson and Lyon 2005; Emberts et al. 2016). Finally, a major morphological factor that is thought to influence autotomy's evolution is an organisms’ body size. Across orthopterans (i.e., grasshoppers, katydids, and allies), for example, larger species autotomize more slowly (Bateman and Fleming 2008). The ease of autotomy might decrease as body size increases because of morphological constraints (discussed in Bateman and Fleming 2008) or differences in predation associated with an organism's size (Remmel et al. 2011).

Leaf‐footed bugs and allies (Insecta: Hemiptera: Coreidae + Alydidae; henceforth referred to as leaf‐footed bugs; see Forthman et al. 2019) are an ideal clade to investigate the evolution of autotomy. These insects autotomize their legs to escape predation (Z. Emberts, pers. obs.), to escape nonpredatory entrapment (Emberts et al. 2016), and to reduce the cost of injury (Emberts et al. 2017a). Species within this clade also show substantial variation in the latency to autotomize their legs (Emberts et al. 2016). In some species, autotomy occurs quickly enough to escape predation (<60 s; Emberts et al. 2016), whereas in other species autotomy takes more than fifteen minutes (Z. Emberts, unpubl. data). Additionally, the autotomizable legs of leaf‐footed bugs come in a variety of forms and serve a variety of functions. Several species have hind legs that resemble their front and mid legs, and legs that take this form are thought to only serve a locomotive function. However, other species have hind legs with enlarged femurs that are costly to develop and maintain for both males and females (Somjee et al. 2018a,b; Joseph et al. 2018; Miller et al. 2019). Given these costs, enlarged hind legs likely have a function beyond locomotion (e.g., some males use them in intrasexual fights over access to females; Miyatake 1997; Mitchell 1980; Eberhard 1998; Miller and Emlen 2010; Miller et al. 2016; Emberts et al. 2017b). Finally, leaf‐footed bugs vary dramatically in body size and have a cosmopolitan distribution.

To investigate the evolution of autotomy in this clade, we quantified the latency to autotomize in 59 species of leaf‐footed bugs from around the world and conducted phylogenetic comparative analyses. For our first aim, we hypothesized that rapid autotomy in leaf‐footed bugs evolved via an intermediate latency step. For our second aim, we hypothesized that predation, the cost autotomy, and body size would all influence the latency to autotomize. Predator diversity and abundance increases toward the equator (Jeanne 1979; Schemske et al. 2009), as do antipredatory traits (Møller and Liang 2013; Díaz et al. 2013; Samia et al. 2015; Levin and York 1978; Laurila et al. 2008). Thus, we predicted that leaf‐footed bugs closer to the equator should autotomize more quickly. We also predicted that species with enlarged hind legs should be less willing to release them because loss of these legs has been shown to decrease an organism's future mating success in some species (Emberts et al. 2018). Finally, we predicted that larger taxa have a slower latency to autotomize, following similar patterns observed in other insect clades (e.g., orthopterans; Bateman and Fleming 2008).

## Methods

### BEHAVIORAL AND MORPHOLOGICAL DATA

To investigate the evolution of autotomy in leaf‐footed bugs, we collected 59 species from the wild, plus one Lygaeidae and two Rhopalidae outgroup taxa (*n* = 62). The species used for this study represent almost all the leaf‐footed bug species that we could find at our field sites in Singapore, Panama, Australia, eSwatini (previously Swaziland), South Africa, and the United States. We ultimately sampled ∼2% of all extant leaf‐footed bug taxa and included species from 15 out of the 40 tribes. Within each species, we aimed to induce autotomy of hind legs in 30 female and 30 male individuals (sample size mean = 20.2, median = 10.5). However, it is important to note that some species are represented by a single individual. We only used individuals that had all their legs (i.e., they had not previously autotomized) because appendage loss has been shown to influence escape decisions (Bateman and Fleming 2006b; Stankowich and Blumstein 2005), including the latency to autotomize a second appendage (Bateman and Fleming 2005; Pears et al. 2018). Autotomy was induced by following a previously established protocol (Emberts et al. 2016) where we gripped the insect's leg with constant pressure (reverse action) forceps while the insect's other legs were in contact with a piece of wood. Time to autotomize was recorded using a stopwatch. We conducted behavioral trials for up to 1 h, and individuals that did not autotomize within this hour were recorded as taking 3600 s to autotomize, a decision that was biologically motivated (Appendix S1). Autotomy trials were conducted between the hours of 7:00 and 23:00 and at a temperature of 27 ± 4°C. We considered a species capable of autotomizing if a single individual dropped their leg in the 1‐h escape from entrapment scenario or if at least one wild caught individual was missing a limb at their autotomy fracture plane (i.e., the morphological plane at which autotomy occurs, which is at the trochanter‐femur joint in this clade). Because every species investigated for this study could autotomize, we quantified the mean and median time to autotomize (two measures of central tendency) for each species and assigned these values for the purposes of trait mapping. All autotomy data were square root transformed to better fit model assumptions (e.g., normality and homoscedasticity). Each individual was also photographed with a scale bar using a digital camera (Canon EOS 50D) so we could measure pronotal width, a body size proxy (Procter et al. 2012), to the nearest micrometer using ImageJ version 1.46 (Abràmoff et al. 2004).

### MOLECULAR DATA AND SEQUENCE ALIGNMENT

Of our 62 taxa, 27 had already been sequenced and aligned (Kieran et al. 2019; Forthman et al. 2019; Forthman et al. unpubl. ms.). For the remaining 35 taxa, genomic DNA was extracted following Forthman et al. (2019) (Forthman et al. unpubl. ms.). Isolated DNA was visualized using 1% agarose gel electrophoresis and quantified using a Qubit 2.0 fluorometer. Samples with DNA concentrations greater than 20 ng/μL were normalized to 10–20 ng/μL. A Biorupter UCD‐300 sonication device (4–10 cycles of 30 s) or a Covaris M220 Focused‐ultrasonicator (20–60 s) was used to fragment high molecular weight samples into 200–1000 bp fragments. For library construction, we used a modified KAPA Hyper Prep Kit protocol, which included the use of iTru universal adapter stubs and 8 bp dual‐indexes (Glenn et al. 2016).

For target enrichment, we used a custom myBaits kit (Arbor Biosciences) that subsampled ultraconserved element (UCE) baits designed by Faircloth (2017). Some samples were subjected to the target enrichment (TE) protocol outlined by Forthman et al. (2019), whereas others were subjected to a touch‐down (TE‐TD) approach (Forthman et al. unpubl. ms.). During touch‐down target enrichment, probes were initially hybridized with library pools at 65°C for 18 h followed by 60°C for 18 h, but this hybridization procedure was terminated prematurely. At the recommendation of Arbor Biosciences, we added additional baits (2.75 μL) to these samples and reran the touch‐down hybridization protocol to completion. Posthybridization washing, amplification, and clean‐up followed Forthman et al. (2019; for TE protocol) or Forthman et al. (unpubl. ms.; for TE‐TD protocol). Enriched pools were quantified using a Qubit 2.0 fluorometer and pooled in equimolar ratios prior to sequencing on an Illumina HiSeq3000 lane (2 × 100) at the University of Florida's Interdisciplinary Center for Biotechnology Research (ICBR).

Sequence reads were demultiplexed and adapters were trimmed from raw sequence reads with illumiprocessor (Faircloth 2013; Bolger et al. 2014). Duplicate reads were filtered using PRINSEQ‐lite version 0.20.4 (Schmieder and Edwards 2011). Remaining reads were error‐corrected with QuorUM version 1.1.0 (Marçais et al. 2015) and de novo assembled in Trinity version 2.8.3 (Grabherr et al. 2011) using default settings. Contigs were matched to UCE probes using PHYLUCE version 1.5.0 (Faircloth 2016). Loci were individually aligned in PHYLUCE using default settings and were internally trimmed using trimAl (Capella‐Gutiérrez et al. 2009). Locus alignments with at least 70% of taxa (567 loci) were concatenated into a supermatrix. PartitionFinder version 2.1.1 (Lanfear et al. 2016) was used to select the best‐fit partitioning scheme (‘rcluster” algorithm; Lanfear et al. 2014) with individual loci treated as data blocks and branch lengths unlinked. All models under the “raxml” option were examined, with model selection based on the corrected Akaike information criterion (AICc). A summary of the newly generated sequence data is given in Table S1.

### PHYLOGENY AND DIVERGENCE TIME ESTIMATION

Phylogenetic reconstruction using maximum likelihood was performed using RAxML version 8.2.3 (Stamatakis 2014). We used GTRGAMMA, performed a partitioned analysis using the output from PartitionFinder (–q), and ran 500 rapid bootstraps (–f a). Divergence time estimation was implemented in BEAST version 1.10.4 (Suchard et al. 2018). Due to the large phylogenomic dataset, we ran our 567 loci through SortaDate (Smith et al. 2018) to reduce alignment complexity and to identify those loci most suitable for dating analysis. Upon completion, we chose the best 50 loci sorted first by bipartition, then root‐to‐tip variance, and then tree length. Adding additional loci has been shown to have a negligible impact on the accuracy of dating analyses (Zheng and Wiens 2015). Priors for our BEAST analysis included GTR + gamma nucleotide substitution model, an uncorrelated relaxed clock, a yule speciation process, constrained species relationships in accordance with our maximum likelihood tree, and four fossil calibration points (see below). We executed five independent Markov chain Monte Carlo (MCMC) chains of 300 million generations and sampled every 10,000 generations. To assess stationarity, effective sample size (ESS), and appropriate burn‐in for each individual chain, we inspected each using Tracer version 1.7.1 (Rambaut et al. 2018). Based on visual inspection of the MCMC chains, we determined that four of five the chains required 25% burn‐in and one of the five chains required 45% burn‐in. These respective burn‐in percentages were used when combining both the log files and tree files. The maximum clade credibility tree was summarized from the remaining sampled trees using TreeAnnotator version 1.10.4, and visualized using FigTree version 1.4.3 (Rambaut 2017).

In addition to our BEAST dating analysis, we also conducted a dating method that uses penalized likelihood as implemented in treePL (Smith and O'Meara 2012), which allowed us to obtain a rapid and reliable validation of our BEAST output, the latter of which was later found to have convergence issues. Fossil placement followed the BEAST analysis with the exception that stem fossils were placed on the nodes immediately preceding their respective stems in the treePL analysis (see below). For this dating method, we first generated 100 bootstrap replicates from our molecular dataset and reconstructed a phylogeny for each using maximum likelihood as implemented in RAxML. When building these trees, we used the topology recovered from the best maximum likelihood tree (Fig. S2) as a constraint to ensure consistent fossil placement for our proceeding step. Next, we dated each of these 100 trees with treePL using a smoothing parameter of 0.1. The range of node ages generated from these 100 time‐calibrated trees were then summarized on to the corresponding nodes of our best maximum likelihood tree (Fig. S2) using TreeAnnotator. We direct the readers to Lu et al. (2018) and Li et al. (2019) for further justification of this method.

For our fossil calibrations, we focused on fossils assigned to the extant genera represented in our phylogeny (*n* = 9) to help ensure accurate identification and placement. Descriptions for each of these fossils were then examined to independently assess their taxonomic assignment. We were confident that four of these fossils were correctly assigned to the Coreoidea superfamily. The fossil described as *Jadera interita* (Cockerell 1909) is indeed a species of Rhopalidae, but we could not confidently assign it to either Rhopalinae or Serinethinae, the two subfamilies. Therefore, we assigned it to the stem of Rhopalidae. The fossil described as *Spartocera insignis* (Heer 1853) has large antenniferous tubercles that are close together and a robust body, superficially resembling *Spartocera batatas*. Because we only have two representatives of *Spartocera* in our phylogeny and because we could not confidently say that it is sister to either of the two species we sampled, we placed this fossil on the stem leading to this clade. The fossil taxon *Homoeocerus attenuatus* (Zhang et al. 1994) was appropriately assigned to the *Homoeocerus* genus given its simple hind legs, short square head with clypeus deflexed between the antenniferous tubercles, antennal segment IV not distinctly longer than segment I, and the humeral angles of the pronotum not prominently expanded, among other traits. Because *Homoeocerus* is not monophyletic (see results), we assigned the fossil to the stem of the clade that minimally included all *Homoeocerus* taxa. Finally, the fossil described as *Alydus pulchellus* (Heer 1853) is referenced as being most similar to the extant species *Hyalymenus tarsatus* (= *Alydus recurvis*). Indeed, the description of its curved hind tibia and humeral spines on the pronotum, along with other characters, suggests that this fossil taxon should be assigned to *Hyalymenus* and not *Alydus*. Because we only have a single extant representative of *Hyalymenus* in our phylogeny, we placed this fossil at the crown node of Alydinae.

*Alydus pulchellus* was collected in Baden‐Württemberg, Germany and *S. insignis* was collected in Rabodoj, Croatia (Heer 1853). Both collecting locations can be placed in the Sarmatian Stage (11.6–12.7 million years ago; Harzhauser and Piller 2004) using stratigraphic dating (Heer 1853). *Jadera interita* was collected in the Green River Formation (Cockerell 1909), which has been estimated to be 48.5–53.5 million years ago by argon isotope dating (Smith et al. 2003). *Homoeocerus attenuatus* was collected in the Shanwang Formation (Zhang et al. 1994), which has been estimated to be 17.3–21.0 million years ago by argon isotope dating (He et al. 2011). We added these four fossil calibrations into both the Bayesian (BEAST) and penalized likelihood (treePL) dating analyses. In BEAST, we applied a lognormal distribution with a mean of five and a standard deviation of one to each of the fossil calibrations discussed. These distributions were then offset by the youngest fossil age reported (i.e., *A. pulchellus* = 11.6 million years ago; *S. insignis* = 11.6 million years ago; *J. interita* = 48.5 million years ago; and *H. attenuatus* = 17.3 million years ago).

### STATISTICAL ANALYSES

**Figure 1:**
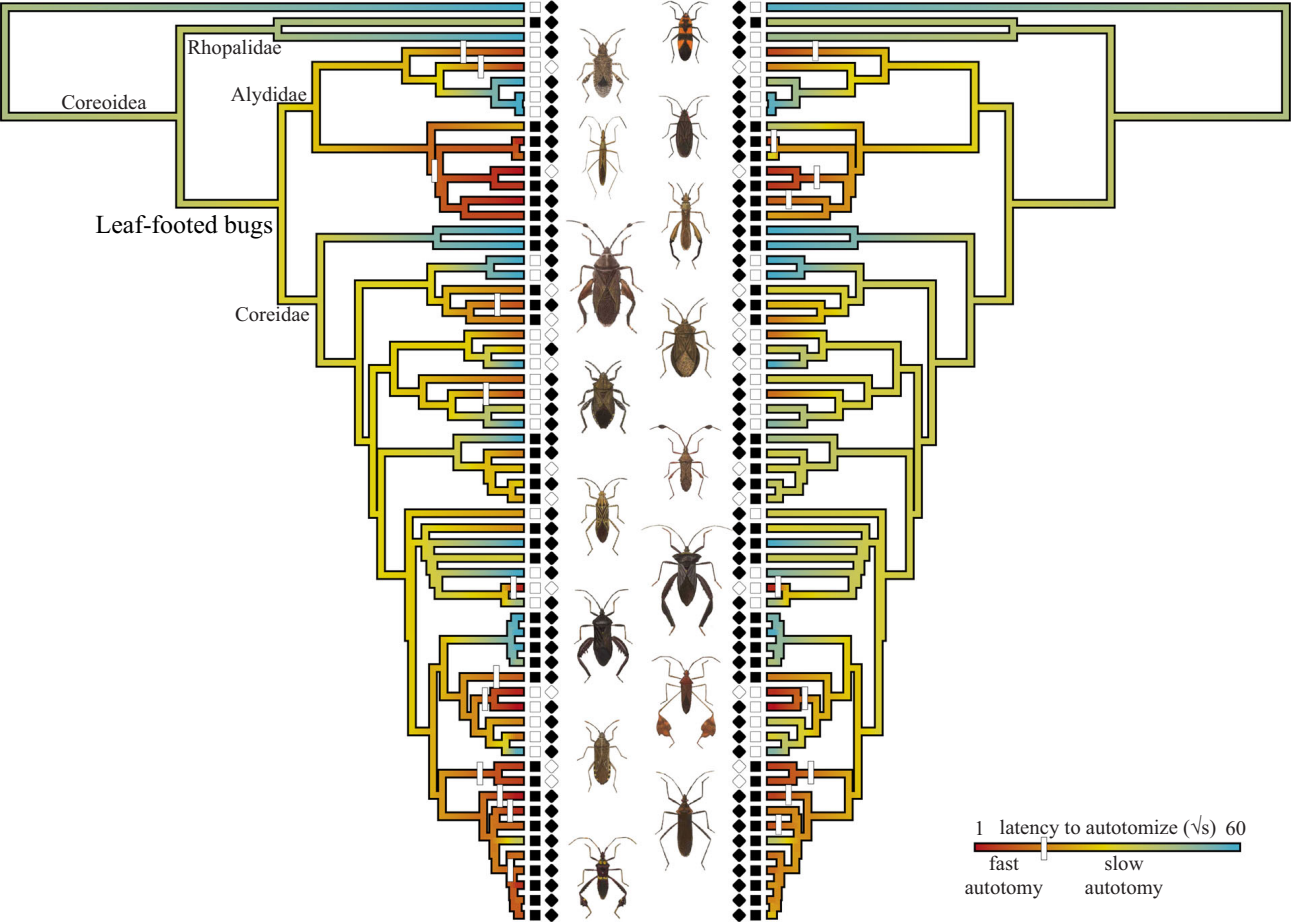
The ancestor of leaf‐footed bugs autotomized their hind limbs slowly. From there, rapid autotomy evolved multiple times (white dashes). Leaf‐footed bugs that autotomize slowly likely use(d) autotomy to reduce the cost of injury or to escape nonpredatory entrapment, but not to escape predation. This suggests that autotomy to escape predation is a co‐opted benefit (i.e., exaptation), revealing one way that sacrificing a limb to escape predation may arise. A visualization of our ancestral state reconstruction illustrates how the median (left) and mean (right) latency to autotomize likely evolved in this clade. Species with enlarged hind leg femurs are represented with filled squares, whereas those with simple hind femurs are represented with open squares. Similarly, species found close to the equator (i.e., within 10 degrees) are represented with open diamonds, whereas those relatively far from the equator (i.e., more than 25 degrees away) are represented with filled diamonds (Fig. S6). Leaf‐footed bug drawings are modified from Distant (1893). See Figure S5 for a visualization of our ancestral state reconstruction that includes tip labels.

To investigate the evolutionary origins of sacrificing a limb to escape predation, we conducted a series of ancestral state reconstructions. For our main analysis, we reconstructed the latency to autotomize using the entire dataset by assigning those that did not drop their leg within an hour as taking 3600 s to autotomize (see Appendix S1). This first analysis carried the implicit assumption that all the individuals investigated in this study would have eventually autotomized their hind legs. We can alternatively assume that individuals who held onto their hind legs for more than an hour could not or would not ever autotomize. Thus, for our second analysis, we reconstructed the latency to autotomize only using individuals we observed autotomizing within an hour (i.e., a subset of the data). This second analysis was then coupled with a third analysis where we reconstructed the proportion of individuals that autotomize within an hour across the tree (e.g., 43 out of 58, 74%, of *Mictis profana* individuals autotomized within an hour, and we wanted to estimate what proportion of the ancestral populations autotomized within an hour). For these analyses, we were particularly interested in estimating the ancestral state at two nodes, the origins of leaf‐footed bugs and coreids more narrowly (Coreidae, per Fig. 1). This approach allowed us to rigorously test our hypotheses on the origins of rapid autotomy. Before conducting any of these analyses, we investigated whether Brownian Motion (BM), Ornstein‐Uhlenbeck (OU), or Early Burst was the best model for our data using geiger version 1.2.2 (Harmon et al. 2008). AICc always identified the best model as an OU model of trait evolution (Table 1). Therefore, we estimated and report on the rate of autotomy for the ancestor of all leaf‐footed bugs and coreids assuming an OU model, as implemented in phytools version 0.6‐60 (Revell 2012). However, we also estimated the rate of autotomy for the ancestor of all leaf‐footed bugs and coreids assuming a BM model of trait evolution because current OU implementations do not specify 95% confidence intervals that enable hypothesis testing. The two models produced quantitatively similar results (BM node estimate = 0.9452 × (OU node estimate) + 2.886; *R* ^2^ = 0.989; Fig. S1). Therefore, we report ancestral state estimations using an OU model of evolution and 95% confidence intervals using a BM model. Because autotomy needs to occur quickly to have an antipredatory benefit and because 120 s is the longest autotomy cutoff that has previously been considered for this capacity (Cooper et al. 2004; Emberts et al. 2019), we reason that if our ancestral state was below 120 s and our 95% confidence interval excluded 120 s, our results would strongly support the fast latency hypothesis. Alternatively, if our ancestor was above 120 s, and our 95% confidence interval excluded 120 s, our results would strongly support the intermediate step hypothesis.

**Table 1:** An OU model of trait evolution best explains how the latency to autotomize evolved

	AICc	AICc	AICc	AICc	AICc	AICc	AICc	AICc	AICc
	all	all	male	male	female	female	autotomy	autotomy	autotomy
	data	data	data	data	data	data	<1 h	<1 h	<1 h
Model	median	mean	median	mean	median	mean	proportion	median	mean
BM	561.0	568.0	517.6	518.0	552.4	551.2	53.3	454.3	450.7
OU	**548.7**	**540.1**	**502.6**	**499.5**	**535.6**	**525.0**	**35.8**	**422.2**	**426.0**
EB	563.2	570.2	519.8	520.3	554.6	553.4	55.5	456.5	453.0

*Note*. Lowest AICc values are bolded.

AICc, corrected Akaike information criterion; BM, Brownian motion; OU, Ornstein‐Uhlenbeck; EB, early burst.

To investigate the factors that contribute to variation in the latency to autotomize across species, we conducted a phylogenetic generalized linear model assuming an OU model of trait evolution as implemented in phylolm version 2.6 (Ho and Ané 2014). We included a body size proxy (i.e., mean pronotal width; continuous), degrees from equator (i.e., the location of the population sampled for this study; continuous), presence of enlarged hind legs (binary), and all pairwise interactions into our full model. Then, we used AICc to reduce model parameters and identify the best model. If two models were tied for the lowest AICc score, then the simpler model was selected as the best model. We also separated our data by sex and reran all statistical analyses to determine whether the importance of a variable was sex specific. All of our latency to autotomize data was square root transformed to better meet model assumptions and then our latency results were back transformed to aid data interpretation.

## Results

### PHYLOGENETIC RELATIONSHIPS

Our maximum likelihood tree found high support (bootstrap values ≥90) for all identified relationships (Fig. S2). These relationships were largely consistent with those previously published given our taxon sampling (Forthman et al. 2019; Forthman et al. unpubl. ms.). For example, our tree also recovered nonmonophyly of the Meropachyinae and Coreinae subfamilies, the Anisoscelini and Hypselonotini tribes, and the *Leptoglossus* genus (Forthman et al. unpubl. ms.). However, our increased taxon sampling of *Homoeocerus* and inclusion of *Mictis* revealed the nonmonophyly of these genera. Specifically, *Prismatocerus auriculatus* was nested within *Homoeocerus*, and *Anoplocnemis* was nested within *Mictis*.

### DATING ANALYSES

Our BEAST dating analysis did not reach an appropriate ESS (i.e., effective sample size >200), despite running for 1,500,000,000 generations. However, visual inspection of the trace suggested that our runs had reached stationarity (i.e., evenness across the trace after burn‐in and a unimodal distribution). Our BEAST analysis placed the origins of the Coreoidea between 51.46 and 54.90 million years ago (median 53.06 million years ago), Coreidae + Alydidae between 24.02 and 49.24 million years ago (median 33.53 million years ago), Coreidae between 23.11 and 40.21 million years ago (median 28.57 million years ago), and Alydidae between 14.07 and 27.84 million years ago (median 18.26 million years ago; Fig. S3). Our treePL dating analysis was largely congruent with the BEAST analysis and placed the origins of Coreoidea at 48.50 million years ago (median 48.50 million years ago), Coreidae + Alydidae between 32.78 and 35.22 million years ago (median 34.00 million years ago), Coreidae between 27.81 and 29.61 million years ago (median 28.49 million years ago), and Alydidae between 28.12 and 29.90 million years ago (median 29.10 million years ago; Fig. S4). Given that both analyses produced similar results (treePL node age = 0.9446 × (BEAST node age) + 3.0734; *R* ^2^ = 0.821) and that our BEAST analysis did not reach an appropriate ESS, all subsequent analyses used the dated tree from treePL (see Appendix S2 for discussion of dating analyses).

### AUTOTOMY

Both the coreid and leaf‐footed bug ancestors autotomized their hind limbs slowly (Fig. 1). Ancestral state reconstructions estimated that 48.3% of the ancestral coreid population and 42.7% of the ancestral leaf‐footed bug population took more than an hour to autotomize their hind limbs. Even those that do autotomize within the first hour were estimated to do so slowly. When we subset the data to only include those that autotomized within an hour, the coreid ancestor was estimated to have a median latency to autotomize of 201 s (95% CI: 3–755 s) and a mean latency to autotomize of 343 s (95% CI: 18–964 s), whereas the leaf‐footed bug ancestor was estimated to have a median latency to autotomize of 157 s (95% CI: <1–729 s) and a mean latency to autotomize of 279 s (95% CI: 44–995 s). When we analyzed all of our data collectively—by assigning those that did not drop their leg within an hour as taking 3600 s to autotomize (see Appendix S1)—the coreid ancestor was estimated to have a median latency to autotomize of 1246 s (95% CI: 207‐3159 s) and a mean latency to autotomize of 1501 s (95% CI: 288–3747 s). Similarly, the leaf‐footed bug ancestor was estimated to have a median latency to autotomize of 1060 s (95% CI: 93–3076 s) and a mean latency to autotomize of 1186 s (95% CI: 137–3624 s). From these slow ancestral rates, autotomizing quickly (<120 s) arose multiple times (Fig. 1). Up to four of these transitions occurred on internal branches, whereas the rest occurred on terminal branches (Fig. 1). These results support the intermediate step hypothesis and in some cases allow us to reject the fast latency hypothesis.

**Figure 2:**
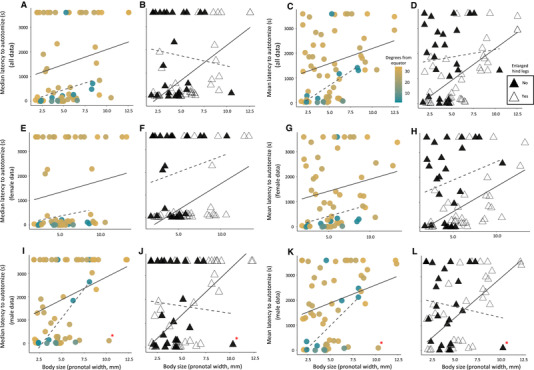
Species that are smaller and closer to the equator autotomize more quickly, and the degree to which having an enlarged hind femur influences the latency to autotomize is sex and size specific. Circle coloration corresponds to distance from the equator, in degrees, from which species were collected (A, C, E, G, I, and K). Because degrees from equator had a bimodal distribution (Fig. S6), we plotted linear regressions associated with each mode to help visualize the data. The solid line includes species relatively far from the equator (>25 degrees), whereas the dashed line includes species closer to the equator (<10 degrees). Note that for a given body size, species closer to the equator often autotomize more quickly. Open triangles and the corresponding solid line regressions denote presence of enlarged hind legs, whereas closed triangles and dashed line regressions correspond to the absence of enlarged hind legs (B, D, F, H, J, and L). Note that for a given body size, species with enlarged hind legs generally autotomize more quickly when analyzing the mean all data (D) and female only data (F and H). However, for the male only data (J and L), there is an interaction between body size and the presence of enlarged hind legs. This interaction is strongly driven by a single data point (Table 2; Fig. S7). Red asterisk denotes deviant data point. Untransformed autotomy data were used in this figure to aid data interpretation. Some data points overlap.

Body size, distance from the equator, and the presence of enlarged hind legs influenced both the median and mean latency to autotomize (Table 2; Figs. 2A‐D). Smaller species, and those closer to the equator, autotomized their limbs more quickly (median, respectively, *t* = 2.464, *P* = 0.017 and *t* = 2.106, *P* = 0.040, and mean, respectively, *t* = 2.932, *P* = 0.005 and *t* = 2.359, *P* = 0.022), as predicted (Figs. 2A and 2C). However, contrary to our predictions, species with enlarged hind legs autotomized more quickly for their given size and locality, although this factor was only statistically significant for the mean latency to autotomize (mean: *t* = –2.090, *P* = 0.041; median: *t* = –1.672, *P* = 0.100; Fig. 2B and 2D). Females returned qualitatively similar results when we separated our data by sex and reran our analyses (Figs. 2E‐H; Tables 2, S2, and S3). The male data, however, were a bit more nuanced (Figs. 2I‐L). The best model for the median male data only included body size and latitude, whereas the best model for the mean male data included all three main effects as well as the pairwise interactions that included body size (Tables 2, S4, and S5). Visual inspection of the male data (Figs. 2I‐L) highlighted the potential for our best models to be driven by a single data point, *Pephricus paradoxus*. Thus, we removed this data point and reran our analysis to determine the sensitivity of the male results. We found that removing this data point resulted in both the median and mean male data converging on the same model, one that included all three main effects as well as the interaction between body size and latitude (Fig. S7; Tables 2, S6, and S7).

**Table 2:** Our model selection criterion (AICc) determined that the best model should include body size, distance from the equator (latitude), and the presence of enlarged hind legs (enlarged leg)

Model	AICc all data median	AICc all data mean	AICc male data median	AICc male data mean	AICc male data minus *Pephricus paradoxus* median	AICc male data minus *Pephricus paradoxus* mean	AICc female data median	AICc female data mean
Latitude	545.3	536.3	509.7	495.0	500.2	484.9	533.7	520.5
Enlarged leg	549.4	540.7	513.5	501.0	504.2	491.1	531.9	523.6
Body size	545.1	536.0	507.5	497.4	495.1	484.9	532.1	522.1
Body size + latitude	542.9	532.9	**506.4**	494.1	493.8	481.2	531.3	518.4
Body size + latitude + body size:latitude	544.4	534.3	506.4	494.2	493.4	481.0	533.1	520.5
Latitude + enlarged Leg	546.3	537.0	511.8	497.0	502.2	486.6	529.6	518.8
Latitude + enlarged leg + latitude:enlarged leg	547.7	538.5	513.3	498.6	503.6	488.0	531.2	520.6
Enlarged leg + body size	544.8	534.6	509.1	498.8	495.6	484.9	524.9	517.5
Enlarged leg + body size + enlarged leg:body size	545.2	536.4	509.1	497.8	497.8	487.1	527.1	519.7
Latitude + enlarged leg + body size	**542.4**	**531.1**	508.2	495.6	494.3	481.0	**523.2**	**512.2**
Latitude + enlarged leg + body Size + enlarged leg:body size	542.4	532.8	507.6	493.7	496.6	483.1	525.5	514.4
Latitude + enlarged leg + body size + latitude:enlarged leg	543.9	532.7	509.8	497.3	495.6	482.4	524.5	513.9
Latitude + enlarged leg + body size + body size:latitude	544.0	532.2	507.9	495.6	**493.3**	**480.3**	525.0	514.4
Latitude + enlarged leg + body size + enlarged leg:body size + latitude:enlarged leg	543.8	534.4	508.5	494.8	497.9	484.5	526.9	516.3
Latitude + enlarged leg + body size + enlarged leg:body size + body size:latitude	543.8	533.9	506.4	**492.5**	495.6	482.2	527.4	516.7
Latitude + enlarged leg + body size + latitude:enlarged leg + body size:latitude	546.0	534.5	510.3	497.9	495.7	482.7	526.8	516.3
Latitude + enlarged leg + body size + latitude:enlarged leg + body size:latitude + enlarged leg:body size	545.8	536.1	509.7	496.2	498.1	484.7	529.3	518.8

*Note*. The male data, after excluding a deviant data point, converged on a model that included these three main effects as well as the interaction between body size and latitude. The best models are bolded. AICc, corrected Akaike information criterion.

## Discussion

Our study clearly indicates that sacrificing a limb to escape predation evolved via an intermediate step in leaf‐footed bugs. All of our analyses supported the conclusion that the leaf‐footed bug ancestor took several minutes to autotomize hind legs. At this rate, the ancestor probably used autotomy to reduce the cost of injury or to escape a fouled molt. Although the ancestor of leaf‐footed bugs autotomized slowly, we found that ∼30% (20 out of 62) of species autotomize quickly enough to use autotomy in an antipredatory capacity (i.e., a latency to autotomize below 120 s). These results suggest that autotomy to escape predation is a co‐opted benefit (i.e., exaptation), revealing one way that sacrificing a limb to escape predation may arise. Our results also highlight that dropping a limb slowly is a pervasive trait found throughout the clade, as more than 30% of the investigated species had a latency to autotomize that was greater than 20 min. Because we have shown that dropping a limb slowly can be an integral step in the evolution of sacrificing a limb to escape predation, future work should continue to quantify the fitness consequences of autotomizing slowly across other clades.

Latitude, body size, and the presence of enlarged hind legs all influence the rate at which species autotomize in this clade. In addition to these main effects, our data from male specimens also reveal the possibility of a body size by latitude interaction as well as a body size by enlarged hind leg interaction. These results provide intriguing insights into the evolution of autotomy.

Species closer to the equator autotomize more quickly, which suggests that leaf‐footed bugs are more likely to use autotomy in an antipredatory capacity when predator diversity and abundance is highest. This result is consistent with other studies that have found a correlation between the strength of predation and the ease of tail autotomy in lizards (Cooper et al. 2004; Brock et al. 2015; Lin et al. 2017, but see Itescu et al. 2017). Moreover, this result agrees with other latitudinal studies, in which antipredatory behaviors are heightened closer to the equator (Møller and Liang 2013; Díaz et al. 2013; Samia et al. 2015). However, because previous studies have mostly investigated FID (i.e., flight initiation distance; Møller and Liang 2013; Díaz et al. 2013; Samia et al. 2015; but Laurila et al. 2008 noted differences in other behaviors), our results highlight the possibility of a more general pattern, that antipredatory *behavior* becomes more relaxed as organisms get farther away from the equator. The idea that antipredatory traits correlate with latitudinal gradients is not new (Schemske et al. 2009), but previous work has mostly focused on morphology (e.g., Vermeij 1978; Palmer 1979).

We also found that larger leaf‐footed bugs autotomize more slowly, which follows a similar pattern observed in another insect clade—orthopterans (i.e., grasshoppers, katydids, and allies; Bateman and Fleming 2008). Although this pattern has been observed before, much remains unknown about the mechanisms that drive this association. There are a few notable differences between larger species of insects when compared to smaller species of insects that could be driving this trend. First, larger species of insects face different predators; larger individuals face more predation by birds and less predation by invertebrates (Remmel et al. 2011). If hind leg autotomy is a less effective antipredatory strategy against birds, for example, then predation may not be selecting for a faster rate of autotomy in larger taxa. Second, as insects get larger, the relative energetic costs of flight may not increase in direct proportion to size (Harrison and Roberts 2000; Niven and Scharlemann 2005). Under certain scenarios, larger insects might rely more on their legs for locomotion, increasing the indirect cost of leg autotomy and constraining its evolution. Finally, larger individuals likely have larger autotomy fracture planes (Z. Emberts, unpubl. data), which may make it harder for them to autotomize (i.e., a morphological constraint). Although body size has been shown to constrain autotomy in orthopterans, and now leaf‐footed bugs, it does not influence autotomy in lizards (Zani 1996). Because lizards can regenerate, whereas orthopterans and leaf‐footed bugs cannot, these differing patterns might be explained by regeneration, which potentially eliminates some constraints on the evolution of autotomy. However, insects and lizards vary in many ways, including the appendage that they autotomize, and the differences in the association between body size and autotomy could be due to any number of factors.

The influence of our latitudinal gradient on the latency to autotomize also becomes less informative as species get larger. This trend is clearly driven by the males (Figs. 2I and 2K; Tables S6 and S7). Such a pattern could be a result of males in larger species being less willing to use autotomy in an antipredatory capacity (i.e., larger species autotomize more slowly). As a result, distance from the equator (i.e., a proxy for the strength of predation) simply explains less variation in the latency to autotomize as taxa get larger. However, this hypothesis itself does not explain why this interaction is sex specific. One possible explanation is that males and females face different susceptibility to predation. In the sweet potato bug, *Physomerus grossipes*—a species included in this study—females guard their eggs against parasitoids (Hemmingsen 1947). Egg guarding in this species confines a female to a single location during reproduction, which may increase the female's chances of becoming prey. We noticed that it made collecting them much easier. Most of these females (70%) autotomized their hind legs within 120 s, despite their large size (mean PW of 6.02 mm). Few males of this species (13%), however, autotomized as quickly. If similar sex differences in predation occur in other large species, this could explain why distance from the equator (i.e., a proxy for the strength of predation) influences latency to autotomize in larger females, but not males.

We also found that leaf‐footed bugs with enlarged hind legs autotomized more quickly, contrary to our hypothesis. Despite the likely costs associated with losing these legs (Emberts et al. 2018), the benefits of autotomy must outweigh the costs in these species. Rapid autotomy of enlarged hind legs may be adaptive for several reasons. First, because the autotomizable appendages are larger, they are likely to have a higher probability of being grabbed by a predator. This would increase selection for these limbs to be dropped quickly. Second, autotomizing larger hind legs may increase the efficacy of autotomy. After an individual successfully uses autotomy in an antipredatory context, the predator then needs to decide whether to continue pursuit of their prey. By autotomizing a larger proportion of their body, prey may ensure that the predators are more content with their meal at hand, and hence reduce the frequency with which the predator continues pursuit. These benefits are not mutually exclusive and either or both of them could outweigh the costs of losing an enlarged hind leg.

Finally, the inclusion of a body size by enlarged hind leg interaction as an explanatory variable for our male autotomy data is the result of a single unique species, *Pephricus paradoxus*. That is to say, excluding this data point removes our body size by hind leg interaction from our best model (Table 2). Despite male autotomy in *P. paradoxus* being a deviant data point, it likely captures biological reality. Much remains unknown about the biology of *P. paradoxus*, but this leaf‐footed bug is closely related to the golden egg bug (*Phyllomorpha laciniata*; i.e., they are both within the Phyllomorphini tribe), a well‐studied species. Golden egg bugs are both morphologically and behaviorally unique. Most notably, *P. laciniata*, together with *P. paradoxus*, has a flared and enlarged pronotum. Because of this unique morphology, pronotal width is likely a poor proxy for their body size relative to other leaf‐footed bugs. In addition to this morphological difference, *P. laciniata* also exhibits paternal care (Reguera and Gomendio 1999; Gomendio and Reguera 2001), which may increase a male's chances of being captured by a predator. This could create increased selection for an antipredator defense that enables males to escape the grasp of a predator, such as autotomy.

The methodology we employed to investigate the evolution of autotomy has strengths as well as limitations. We initially believed that our sampling, which included two species from Rhopalidae and one species from Lygaeidae (taxa outside of leaf‐footed bugs), would recover the evolutionary origins of autotomy. However, every taxon met our criteria for having the ability to autotomize. This could have been problematic if we found support for the fast latency hypothesis (i.e., if the leaf‐footed bug ancestor had been reconstructed as autotomizing in less than 120 s) because it would have been unclear whether a more distant ancestor could have autotomized slowly. In that case, we would have been unable to reject the intermediate step hypothesis. Another potential issue with our taxon sampling was that it led to discontinuity in our sampling of distance from the equator. Although having taxa evenly sampled across our latitudinal gradient would have been ideal (as numerous leaf‐footed bugs can be found between 10 and 25 degrees from the equator), our bimodal sampling is not problematic for our statistical analyses (i.e., our analyses met linear model assumptions), nor our biological interpretation (i.e., there were species close to the equator and far from the equator). Additionally, we do not know the mechanistic causes of the patterns that we have shown here. For example, we found that distance from the equator explained variation in the latency to autotomize and we postulate that this is driven by predation. However, it is possible that other factors that correlate with latitude may be responsible for this trend, such as seasonality of temperature. Now that these associations have been identified, more focused studies should identify the mechanism behind them. Finally, to test our alternative hypotheses on the origins of rapid autotomy, we had to assign a cutoff value; in this case, we selected 120s. Although 120s may seem like a long time for a predator‐prey interaction, we a priori selected this time point because (1) the mean values of time‐to‐event data can overestimate a distributions central tendency when it has a right skew, (2) it is the longest time cutoff that has previously been used (Cooper et al. 2004; Emberts et al. 2019), and (3) it made biological sense given the predators and mobility of leaf‐footed bugs. Even if one decided that a lower threshold such as 60s or 10s would be more appropriate, our results would still qualitatively hold because our 95% confidence intervals excluded these values.

Autotomy is one of the most extreme forms of antipredatory defense, dramatically illustrating the importance of survival in the context of natural selection. Nonetheless, fundamental questions of how this extreme trait evolves have remained unanswered. Here, we show that some leaf‐footed bugs evolved the ability to autotomize their limbs rapidly enough to escape predation via an intermediate latency step. However, this is just one possible pathway by which rapid autotomy may evolve and future studies should investigate how autotomy has evolved in other clades (e.g., walking sticks, salamanders, decapod crustaceans, spiders, and harvestmen). Moreover, future studies should seek to investigate the evolutionary origins of autotomy itself as opposed to the origins of autotomizing quickly. Studies that investigate the evolutionary morphology of autotomy fracture planes and/or the evolutionary physiology of autotomy would provide valuable insights. Our study also highlights the possibility of a broad latitudinal pattern—that antipredatory behaviors become more relaxed farther away from the equator. Future work should continue to investigate this association with autotomy, as well as other antipredatory behaviors, such as vigilance and death feigning. Such studies will continue to shed light on the factors that contribute to the behavioral diversity in antipredator responses observed throughout Animalia.

Associate Editor: G. Hobel

Handling Editor: T. Chapman

## Supporting information


**Appendix S1**. Individuals that did not autotomize within one hour were recorded as taking 3600 s to autotomize.
**Appendix S1 Figure 1**. Histogram of the latency to autotomize for *Micitis profana* when using a 3600 s escape from entrapment scenario (A), compared to a 2000 s escape from entrapment scenario (B).
**Appendix S2**. Our dating analyses revealed younger age estimates than previously reported.
**Appendix S3**. Both male and female leaf‐footed bug ancestors autotomized their hind legs slowly.
**Figure S1**. Comparing ancestral state reconstructions when assuming an Ornstein‐Uhlenbeck (OU) model of trait evolution to a Brownian Motion (BM) model of trait evolution for the all data combined dataset.
**Figure S2**. RAxML best tree with bootstrap values labeled at the nodes.
**Figure S3**. Dated BEAST tree with median node ages labeled and bars denoting the 95% highest probability density interval.
**Figure S4**. Dated TreePL tree with median node ages labeled and error bars that show the range of age estimates across 100 bootstrap trees.
**Figure S5**. Ancestral state reconstruction for the median (left) and mean (right) latency to autotomize (i.e., Figure 1) with tip labels.
**Figure S6**. Degrees from equator, our latitudinal gradient, had a bimodal distribution.
**Figure S7**. Small males with enlarged hind legs found near the equator autotomize quickly.
**Table S1**. Summary data for sequence reads, contigs, and ultraconserved element loci generated in this study.
**Table S2**. Best model for the median female data.
**Table S3**. Best model for the mean female data.
**Table S4**. Best models for the median male data
**Table S5**. Best model for the mean male data.
**Table S6**. Best model for the mean male data, minus *Pephricus paradoxus*.
**Table S7**. Best model for the median male data, minus *Pephricus paradoxus*.
