# Supplementary material for: The evolution of autotomy in leaf‐footed bugs
Source: Evolution. 2020 Apr 8;74(5):897–910. doi: 10.1111/evo.13948 (PMC7317576; doi:10.1111/evo.13948)
Supplement: Supplementary file 1 — Appendix S1. Individuals that did not autotomize within one hour were recorded as taking 3600 s to autotomize. Appendix S1 Figure 1. Histogram of the latency to autotomize for Micitis profana when using a 3600 s escape from entrapment scenario (A), compared to a 2000 s escape from entrapment scenario (B). Appendix S2. Our dating analyses revealed younger age estimates than previously reported. Appendix S3. Both male and female leaf‐footed bug ancestors autotomized their hind legs slowly. Figure S1. Comparing ancestral state reconstructions when assuming an Ornstein‐Uhlenbeck (OU) model of trait evolution to a Brownian Motion (BM) model of trait evolution for the all data combined dataset. Figure S2. RAxML best tree with bootstrap values labeled at the nodes. Figure S3. Dated BEAST tree with median node ages labeled and bars denoting the 95% highest probability density interval. Figure S4. Dated TreePL tree with median node ages labeled and error bars that show the range of age estimates across 100 bootstrap trees. Figure S5. Ancestral state reconstruction for the median (left) and mean (right) latency to autotomize (i.e., Figure 1) with tip labels. Figure S6. Degrees from equator, our latitudinal gradient, had a bimodal distribution. Figure S7. Small males with enlarged hind legs found near the equator autotomize quickly. Table S1. Summary data for sequence reads, contigs, and ultraconserved element loci generated in this study. Table S2. Best model for the median female data. Table S3. Best model for the mean female data. Table S4. Best models for the median male data Table S5. Best model for the mean male data. Table S6. Best model for the mean male data, minus Pephricus paradoxus. Table S7. Best model for the median male data, minus Pephricus paradoxus. [file EVO-74-897-s001.pdf]

Supplementary file for:

The evolution of autotomy in leaf-footed bugs

Authors: Zachary Emberts[1*], Colette M St. Mary[1], Cody Coyotee Howard[1, 2], Michael Forthman[3], Philip W Bateman[4], Ummat Somjee[5], Wei Song Hwang[6], Daiqin Li[7], Rebecca T Kimball[1], Christine W Miller[3]

Affiliations:

[1] Department of Biology, University of Florida, 876 Newell Drive Gainesville, Florida 32611, USA

[2] Florida Museum of Natural History, University of Florida, Gainesville, Florida 32611, USA

[3] Entomology & Nematology Department, University of Florida, 1881 Natural Area Drive Gainesville, Florida 32611, USA

[4] Behavioural Ecology Lab, School of Molecular and Life Sciences, Curtin University, Bentley, Perth WA 6845, Australia

[5] Smithsonian Tropical Research Institute, Balboa, Ancón, Panamá

[6] Lee Kong Chian Natural History Museum, National University of Singapore, 2 Conservatory Drive, Singapore 117377, Singapore

[7] Department of Biological Science, National University of Singapore, 14 Science Drive 4, Singapore 117543, Singapore

*Correspondence to: emberts@ufl.edu

Appendix S1. Individuals that did not autotomize within one hour were recorded as taking 3600 s to autotomize.

To investigate the evolution of autotomy in leaf-footed bugs we conducted 1,253 autotomy trials. During these trials, individuals were given 1 hour (3600 s) to escape from entrapment. Although a majority of individuals autotomized their hind limb during this scenario, 498 did not. Lack of autotomy during the given timeframe can be interpreted in one of two ways. First, it is possible that these individuals cannot or will not autotomize their hind legs. Second, it is possible that these individuals would have autotomized their hind legs if they were given more time. Although we cannot rule out the first hypothesis completely, we do have evidence to suggest that many, if not most, of these individuals would have eventually autotomized their hind legs.

Evidence suggests that all the leaf-footed bugs included in this study can autotomize. Of the 62 species investigated, we provide data showing that 56 of them can drop their legs. Of the remaining 6 species, we have personally observed *Spartocera fusca*, *Stenocoris filiformis*, *Acanthocephala thomasi*, *Piezogaster calcarator*, and *Homoeocerus angulatus* autotomizing so we are confident that they can autotomize their legs as well. It is worth noting that we have not observed *Spartocera batatas* autotomizing. However, we have observed wild caught *S. batatas* missing legs at their trochanter-femur joint (i.e., the autotomy fracture plane). Thus, we have no reason to believe that any leaf-footed bug species is incapable of autotomy.

There is also strong evidence to suggest that some leaf-footed bugs will wait for more than an hour before autotomizing an entrapped limb. In a different study (Z.E. unpublished data), we gave individuals 2 hours to escape from entrapment. Only 5 of the 24 *Acanthacephala declivis* investigated in the study autotomized their hind leg within 1 hour, while 9 individuals autotomized their hind leg within 2 hours. That is to say, 4 additional individuals took between 1 and 2 hours to autotomize their hind leg. This data nicely illustrates why it is reasonable for us to assume that more leaf-footed bugs would have autotomized their hind legs if we had given them more time. We do acknowledge that some individuals investigated in this study may have never autotomized their hind legs. It is conceivable, for example, that some individuals may not have had enough energy to perform autotomy (i.e., a physiological constraint). However, such scenarios are likely the exception and not the rule.

Given that autotomy can occur after one hour, we decided to record individuals that did not autotomize during the study as taking 3600 s to autotomize. This approach will underestimate the true mean of the sampled population if we reasonably assume that all of the individuals would have eventually autotomized (i.e., taken longer than 3600 s to autotomize). Consequently, our finding that the ancestor of leaf-footed bugs took 19 minutes to autotomize their hind leg on average is likely an underestimation, which strengthens our conclusion that the ancestor autotomized slowly. The alternative to assigning those that did not drop their leg as taking 3600 s to autotomize would be to exclude these individuals from our analyses. Removal of these data points would require us to assume that all of these individuals cannot or will not autotomize, an unrealistic assumption. This could result in us considerably underestimating a species true latency to autotomize.

Using median latency to autotomize ameliorates most of the concerns about how we should deal with individuals that do not autotomize. Specifically, if at least half of the individuals investigated per species autotomized within 1 hour, our results would accurately reflect the true median of the sampled population. This occurred in 70% of the investigated species (44 out of 62). The robustness of median can be illustrated nicely using our data on the

latency to autotomize in *Mictis profana* (Appendix S1 Figure 1). Image a scenario in which we gave individuals 2000 s to escape from entrapment, as compared to 3600 s. Under the 2000 s scenario, we would have observed 38 individuals autotomizing their entrapped leg, and 20 that retained them (which we assign as taking 2000 s to autotomize). The mean and median of the data under this scenario would be 842 and 256 s, respectively. If we compare these results to our 3600 s escape from entrapment scenario (i.e., our actual results) we find that 43 individuals autotomized and 15 did not (which we assign as taking 3600 s to autotomize). The mean and median for this data is 1,321 and 256 s, respectively. Note that the median is 256 s for both scenarios. In addition to addressing concerns about how to deal with individuals that did not autotomize, median is also a better measure of central tendency for species that have a right skewed distribution with a long tail, as is the case for *M. profana* (Appendix S1 Figure 1).

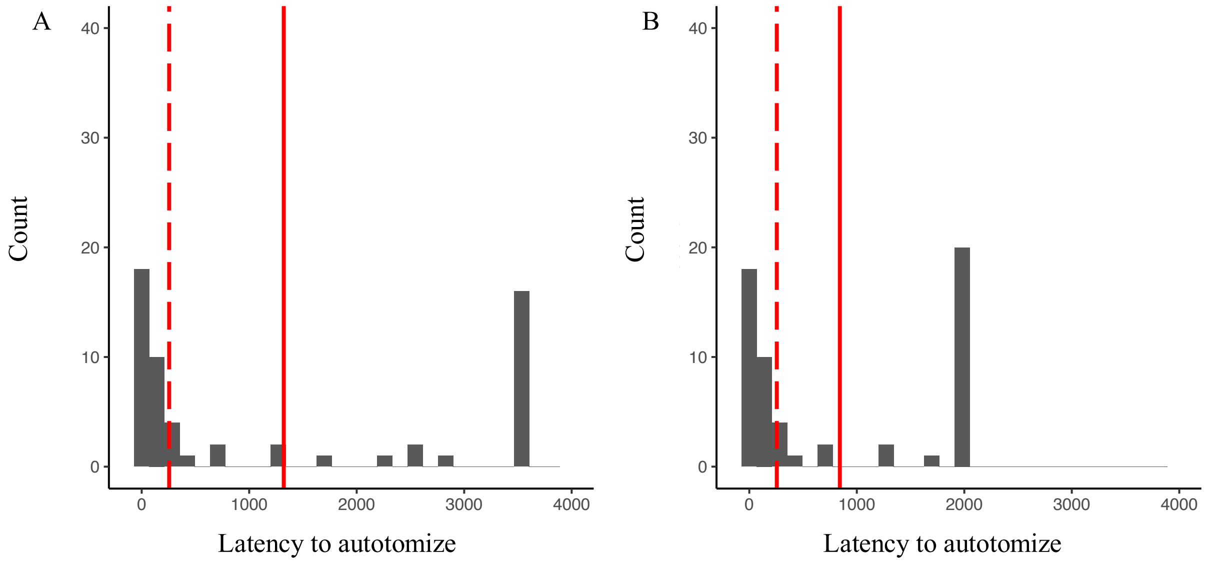

Appendix S1 Figure 1. Histogram of the latency to autotomize for *Micitis profana* when using a 3600 s escape from entrapment scenario (A), compared to a 2000 s escape from entrapment scenario (B). Note that the distribution for the latency to autotomize has a right skew, which is common for time-to-event data. Dashed lines represent medians, while solid lines represent means. In this context, our mean result is sensitive to our chosen escape from entrapment scenario while our median result is not because more than 50% of the individuals autotomized.

Appendix S2. Our dating analyses revealed younger age estimates than previously reported.

Our BEAST dating analysis placed the origins of the Coreoidea between 51.46–54.9 mya (median 53.06 mya) and Coreidae + Alydidae between 24.02–49.24 mya (median 33.53 mya). Whereas our treePL dating analysis placed Coreoidea at 48.5 mya (median 48.5 mya) and Coreidae + Alydidae between 32.7785–35.2195 mya (median 33.9972 mya). Both dating analyses identify these clades as originating more recently than previously reported (Wang et al. 2016, Li et al. 2017, Johnson et al. 2018, Liu et al. 2019). Wang et al. (2016) found Coreoidea originated around 153-166 mya (median 160) and Coreidae + Alydidae around 112–154mya (median 138). Wang et al. (2016) used BEAST to date their tree and included 15 fossil calibrations. Li et al (2017) estimated the origin of Coreoidea to be 157 mya (range 143–168) and Liu et al (2019) estimated the Coreoidea origin to be 162 mya (range 139–179); both studies dated their trees with PhyloBayes. Finally, Johnson and colleagues (2018) estimated Coreoidea to have originated around 93 mya and Coreidae + Alydidae around 72 mya using MCMCTree (Bayesian) molecular dating analysis as implemented in PAML. Differences between our age estimates and those previously reported are most likely a result of using different fossil calibrations. None of the fossil calibrations in these other studies included Coreidae or Alydidae fossils, while our study included three. Moreover, differences in the specific dating analyses and their specified parameters, taxon sampling, and differences in how the molecular data were obtained likely contribute to variation in the age estimates as well.

Appendix S3. Both male and female leaf-footed bug ancestors autotomized their hind legs slowly.

Ancestral state reconstruction using only the male data estimated the ancestor of leaf-footed bugs to have a median latency to autotomize of 1,419 s (back transformed from 37.67 √s) under both an OU and BM model of trait evolution, with a 95% confidence interval that ranged from 178–3,843 s (back transformed from 13.343–61.993 √s). For the female data, the ancestor of leaf-footed bugs was estimated to have a median latency to autotomize of 894 s (back transformed from 29.893 √s) assuming an OU model of trait evolution and 899 s (back transformed from 29.979 √s) when assuming BM. The 95% confidence interval for the female leaf-footed bug ancestor ranged from 9–3,236 s (back transformed from 3.069–56.890 √s).

Ancestral state reconstruction using only the male data estimated the ancestor of leaf-footed bugs to have a mean latency to autotomize of 1,552 s (back transformed from 39.396 √s assuming an OU model of trait evolution and 39.398 √s assuming a BM model) with a 95% confidence interval that ranged from 289–3,820 s (back transformed from 16.993–61.804 √s assuming a BM model of trait evolution). With only the female data the ancestor of leaf-footed bugs is estimated to have a mean latency to autotomize of 947 s (back transformed from 30.779 √s) assuming an OU model of trait evolution and 1,024 s (back transformed from 31.995 √s) when assuming a BM model of trait evolution. The 95% confidence interval for the leaf-footed bug ancestor using the female data ranges from 29–3,437 s (back transformed from 5.361–58.629 √s) under BM.

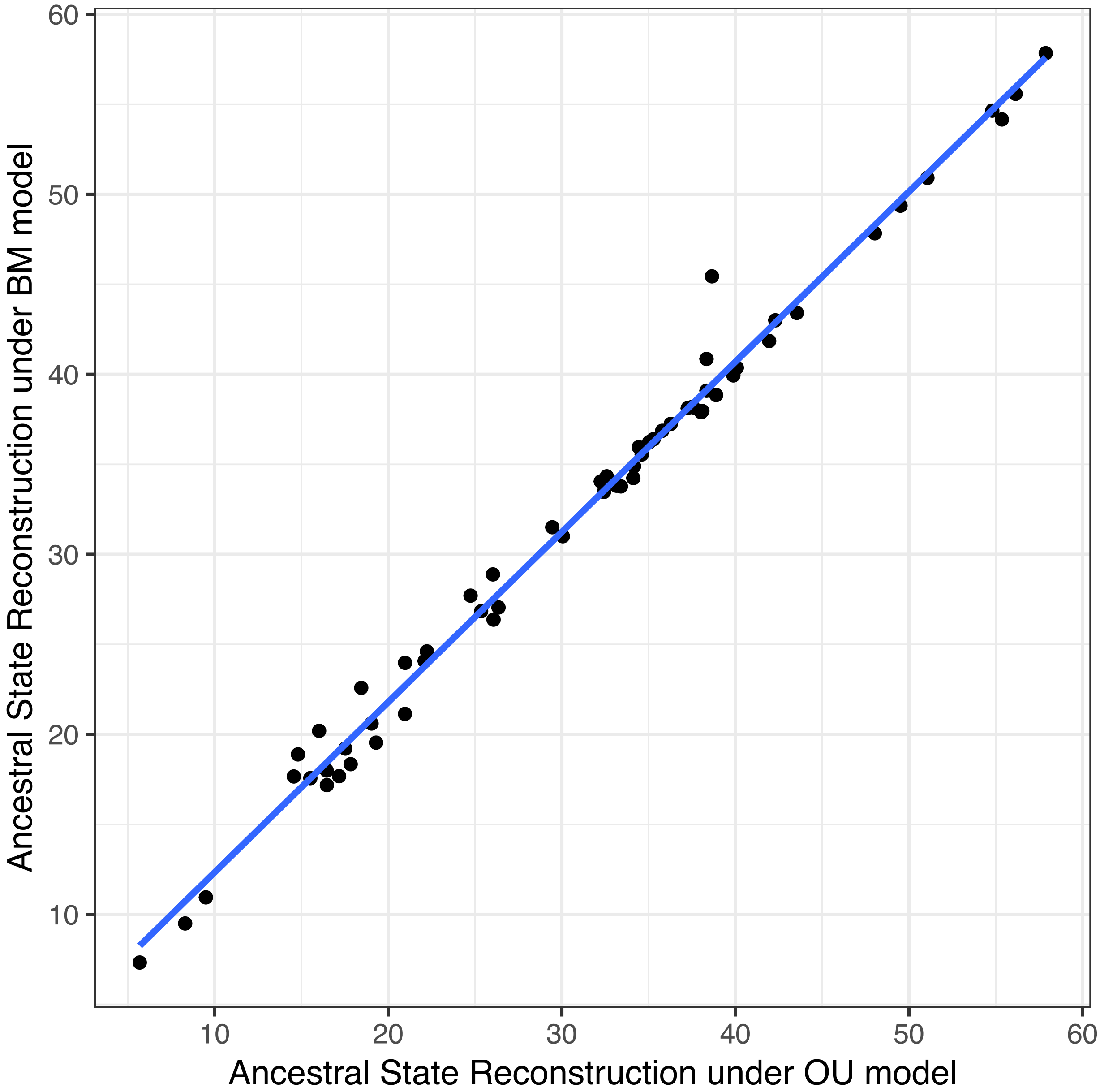

Figure S1. Comparing ancestral state reconstructions when assuming an Ornstein-Uhlenbeck (OU) model of trait evolution to a Brownian Motion (BM) model of trait evolution for the all data combined dataset. The OU estimates slightly quicker rates of autotomy (based on square root transformed mean latency to autotomize in seconds) at ancestral nodes.

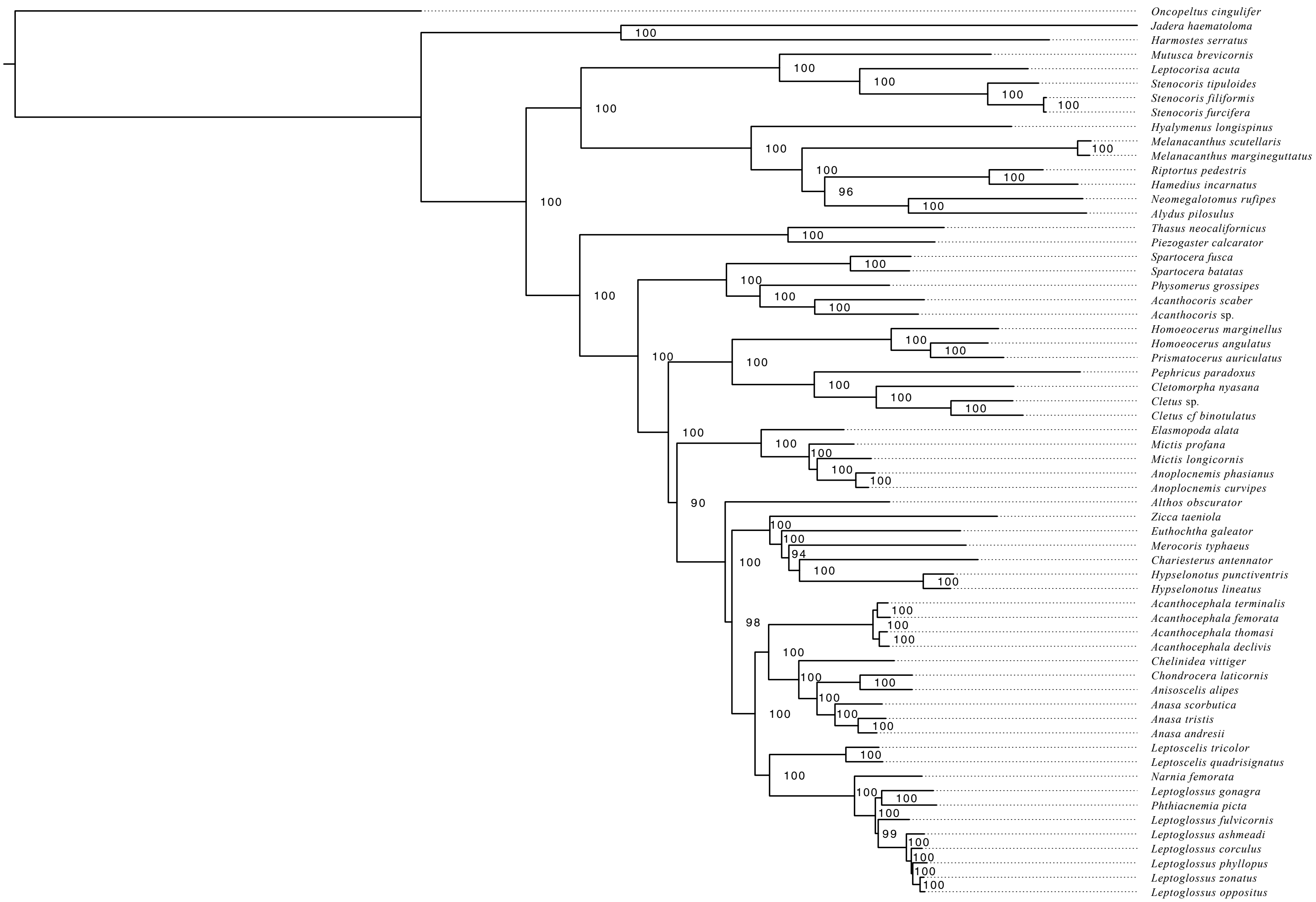

Figure S2. RAxML best tree with bootstrap values labeled at the nodes.

Emberts et al. 2020. *Evolution*.

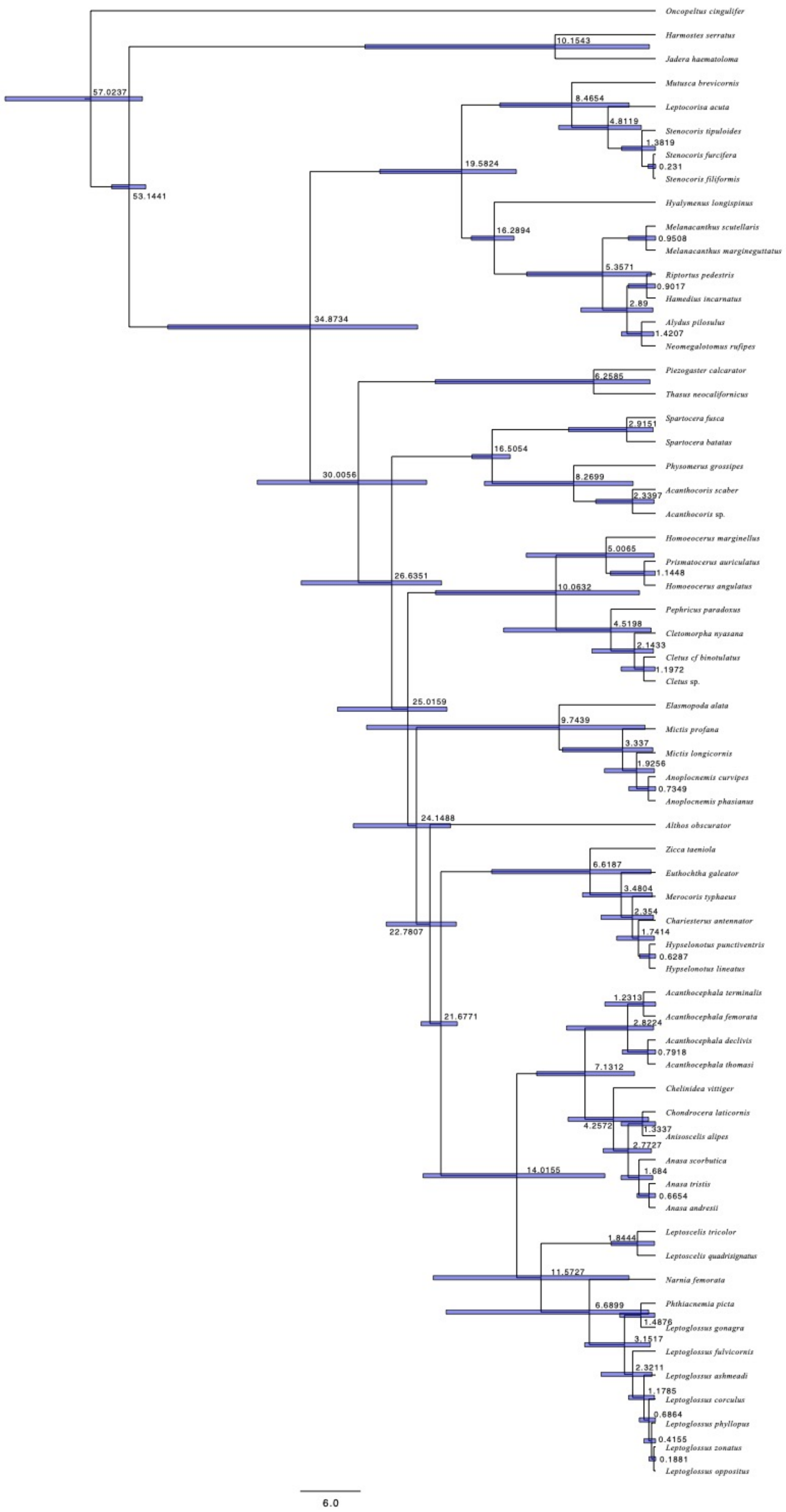

Figure S3. Dated BEAST tree with median node ages labeled and bars denoting the 95% highest probability density interval.

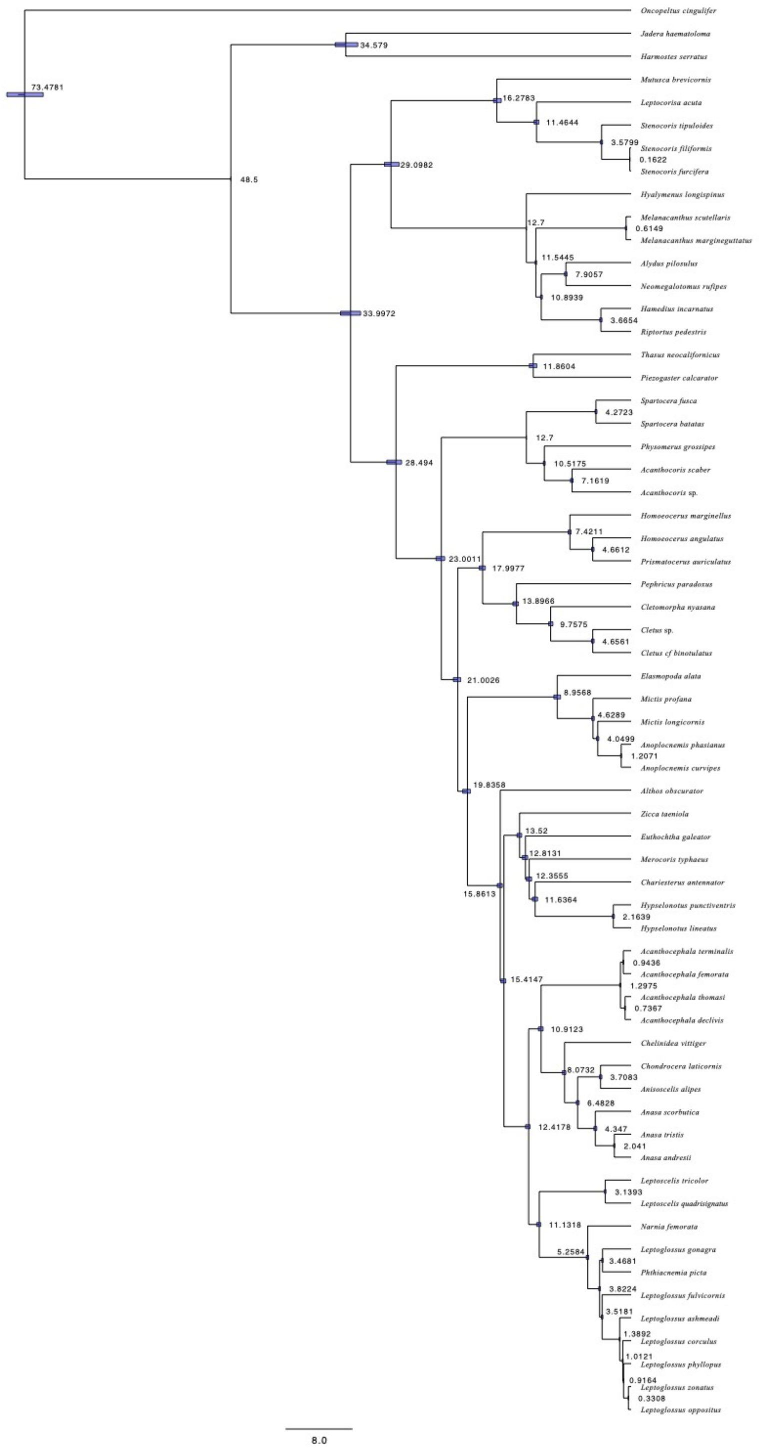

Figure S4. Dated TreePL tree with median node ages labeled and error bars that show the range of age estimates across 100 bootstrap trees. An absent error bar at a node means that all 100 bootstrap trees converged on the same age for that node.

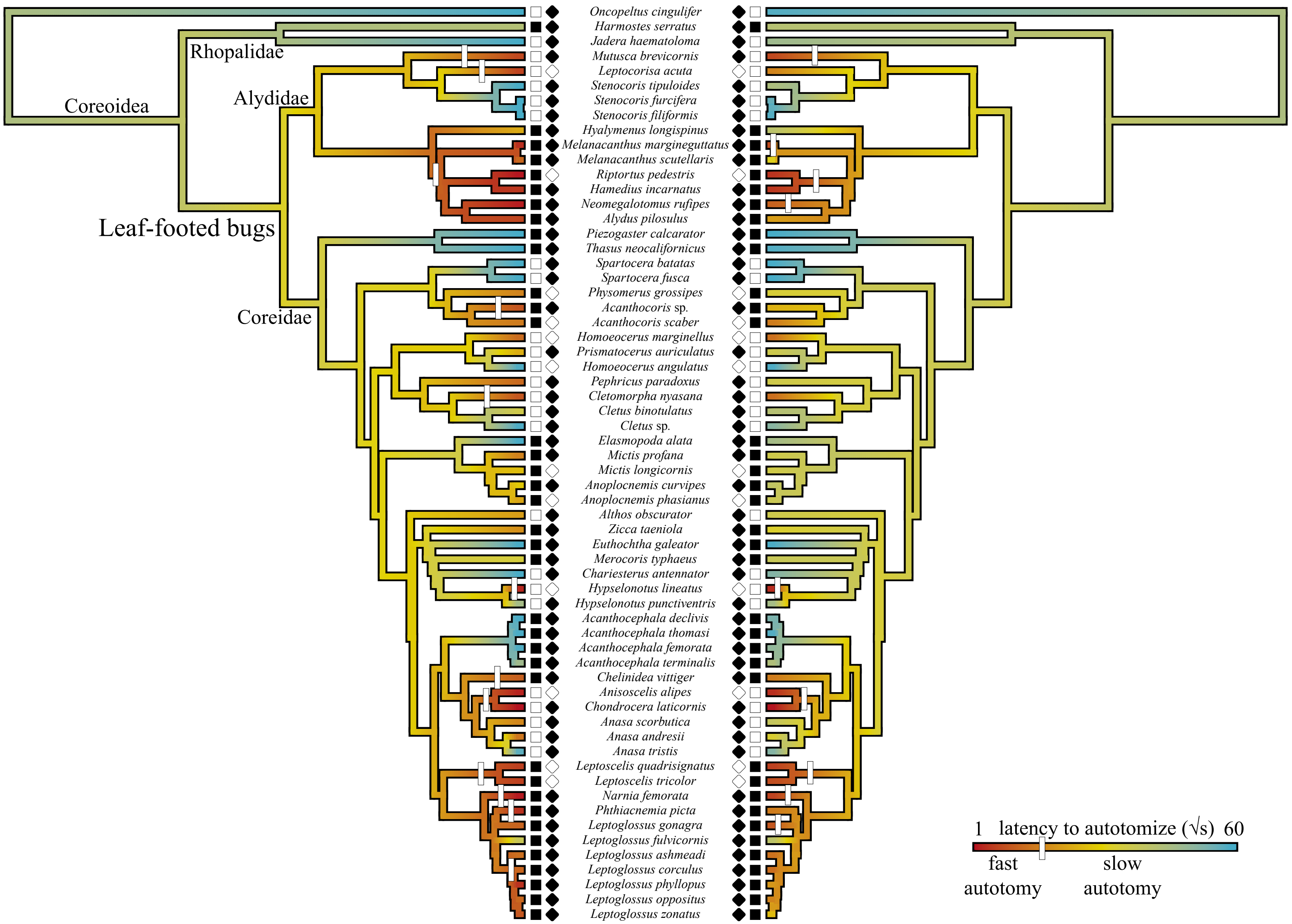

Figure S5. Ancestral state reconstruction for the median (left) and mean (right) latency to autotomize (i.e., Figure 1) with tip labels.

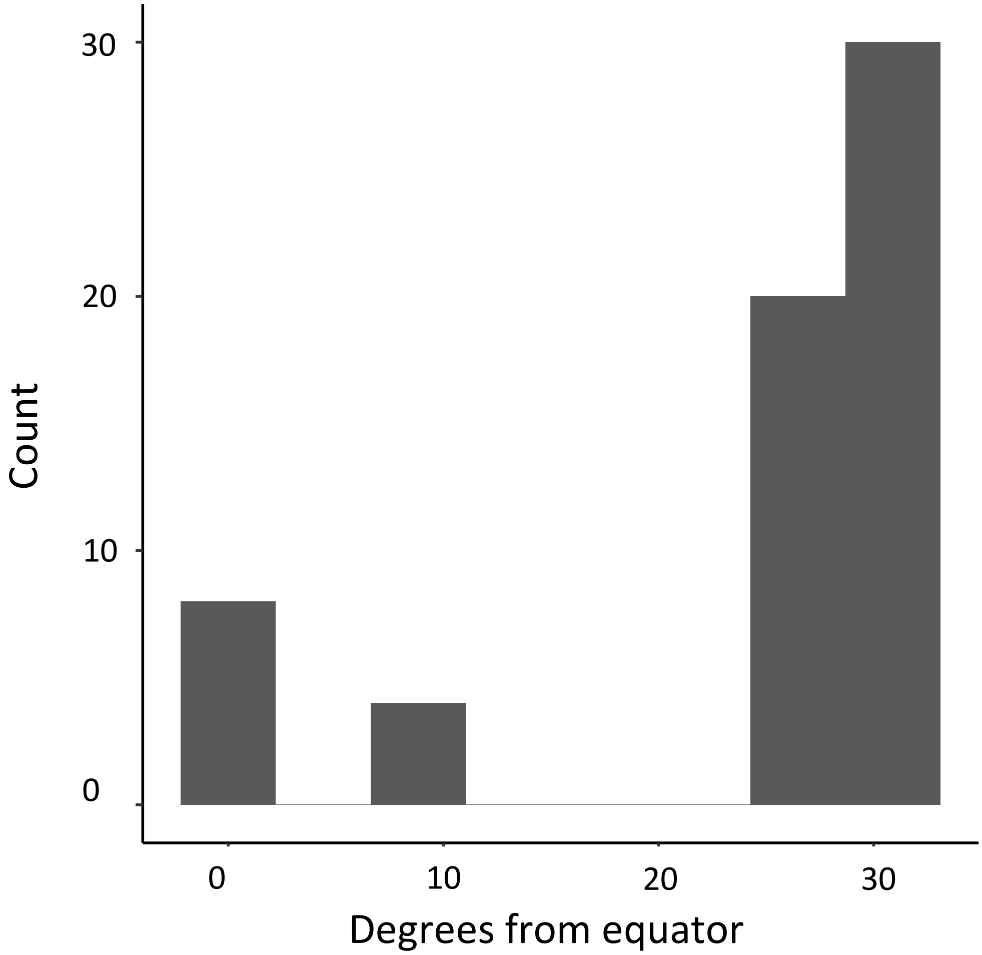

Figure S6. Degrees from equator, our latitudinal gradient, had a bimodal distribution. Therefore, we categorized species that were collected within 10 degrees of the equator as species close to the equator and those farther than 25 degrees away from the equator as species far from the equator. This categorization was *only* used to help visualize the potential interaction between body size and degrees from the equator in Figure 1 and 2, and not used in statistical analyses.

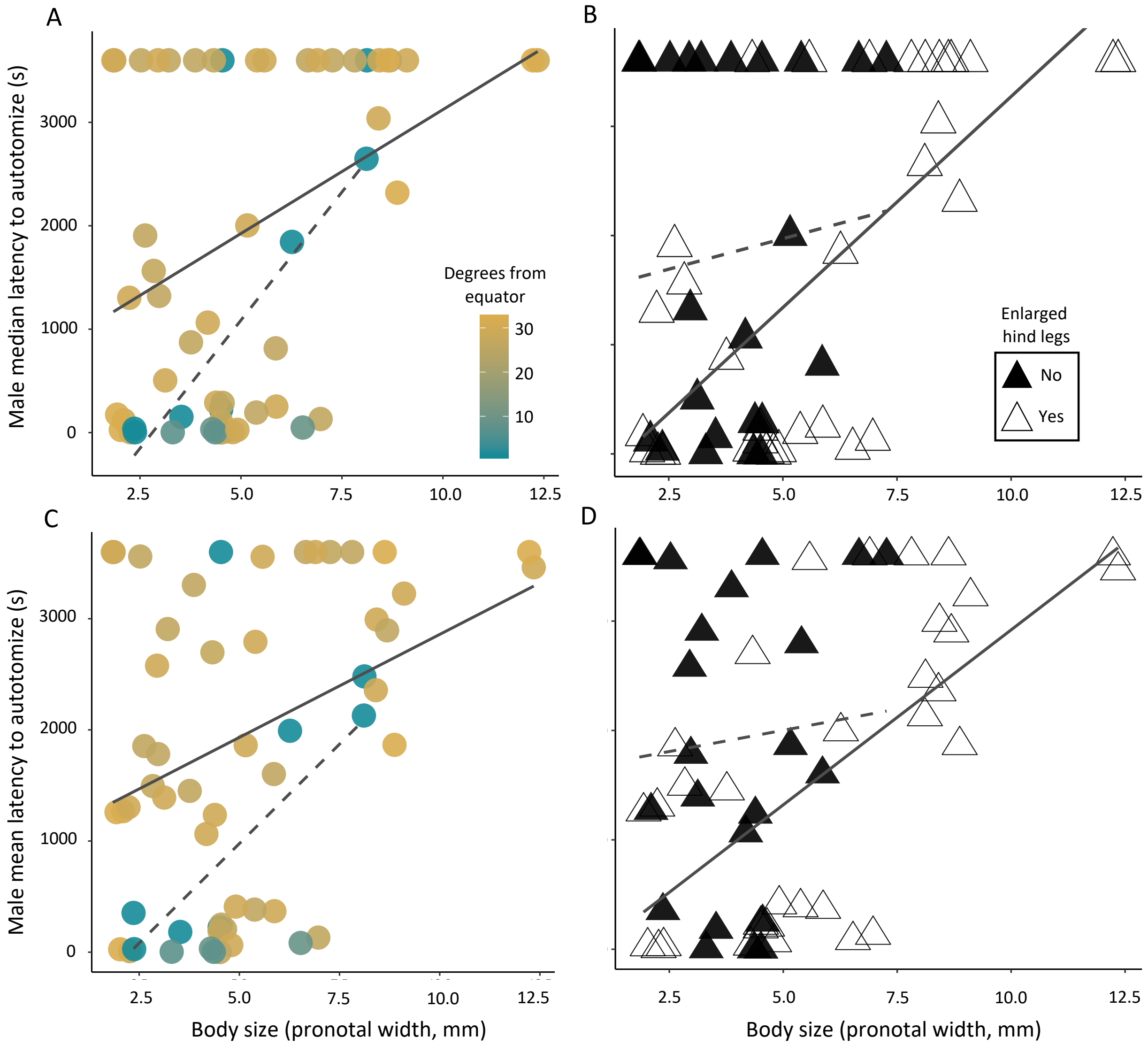

Figure S7. Small males with enlarged hind legs found near the equator autotomize quickly. Here, we visualize the male autotomy data while removing a single data point (*Pephricus paradoxus*, which has a median latency to autotomize of 114 s [A and B] and mean of 97 s [C and D], a body size of 10.3 mm, was collected 26.3 degrees away from equator, and does not have enlarged hind legs). Notice that the interactions are not as distinct after removing our *P. paradoxus* data point when compared to Figure 2I-L. Circle coloration corresponds to distance from the equator, in degrees, from which species were collected (A and C). Open triangles and the corresponding solid lined regressions denote presence of enlarged hind legs, while closed triangles and dashed lined regressions correspond to the absence of enlarged hind legs (B and D). Untransformed autotomy data was used in this figure to aid data interpretation.

Family	Subfamily	Tribe	Genus	Species	Paired Reads	Reads passed QC	Contigs	Total bp	Mean contig length	Min contig length	Max contig length	UCE loci	% UCE recovered	Mean UCE length	Min UCE length	Max UCE length
Alydidae	Alydinae		*Alydus*	*pilosulus*	7444142	4599199	20362	9519217	467.50	181	5593	1278	47.81	760.2042254	202	3380
Alydidae	Alydinae		*Melanacanthus*	*margineguttatus*	8421192	4104664	9967	5309317	532.69	188	4191	778	29.11	729.8020566	201	3089
Alydidae	Alydinae		*Melanacanthus*	*scutellaris*	5436566	2544625	6848	3388540	494.82	193	3329	780	29.18	605.2230769	201	2646
Alydidae	Alydinae		*Riptortus*	*pedestris*	4581022	2956378	16876	8440804	500.17	194	12071	1252	46.84	425.4464856	201	2775
Alydidae	Micrelytrinae	Leptocorisini	*Leptocorisa*	*acuta*	8562652	4386954	20715	8378223	404.45	197	2996	1339	50.09	652.8170276	201	2548
Alydidae	Micrelytrinae	Leptocorisini	*Stenocoris*	*filiformis*	4779514	2787529	15624	6718365	430.00	200	5275	1463	54.73	633.7382092	201	2701
Alydidae	Micrelytrinae	Leptocorisini	*Stenocoris*	*furcifera*	13734054	4350648	17991	6563582	364.83	201	2845	1499	56.08	566.8925951	202	2543
Coreidae	Coreinae	Acanthocephalini	*Acanthocephala*	*delcivis*	5535530	2273749	6117	3047522	498.21	188	2835	920	34.42	695.7086957	201	2835
Coreidae	Coreinae	Acanthocephalini	*Acanthocephala*	*terminalis*	7401210	3257152	7158	3630523	507.20	201	3296	954	35.69	726.0178197	201	2983
Coreidae	Coreinae	Acanthocorini	*Acanthocoris*	*scaber*	4065822	1651257	7141	3154704	441.77	201	2620	947	35.43	561.5142555	201	2620
Coreidae	Coreinae	Acanthocorini	*Acanthocoris*	sp.	6599656	3025631	21830	9700951	444.39	191	5171	1388	51.93	728.3436599	201	2955
Coreidae	Coreinae	Acanthocorini	*Physomerus*	*grossipes*	4025938	2173257	8080	3468458	429.26	183	4375	719	26.90	681.5577191	201	4375
Coreidae	Coreinae	Anisoscelini	*Leptoglossus*	*ashmeadi*	5926698	3085191	8061	3685247	457.17	190	3505	825	30.86	698.2715152	202	2810
Coreidae	Coreinae	Anisoscelini	*Leptoglossus*	*corculus*	4279664	2569863	16452	7005204	425.80	185	3625	1261	47.18	681.0150674	201	3279
Coreidae	Coreinae	Anisoscelini	*Leptoglossus*	*fulvicornis*	4425804	2221638	12537	4661345	371.81	191	3041	1205	45.08	425.2207469	201	2328
Coreidae	Coreinae	Anisoscelini	*Leptoglossus*	*oppositus*	5816802	2719254	8089	3522975	435.53	199	2847	811	30.34	609.0567201	201	2847
Coreidae	Coreinae	Anisoscelini	*Leptoglossus*	*zonatus*	6491074	3334668	11091	5559863	501.30	184	4236	969	36.25	829.120743	201	3255
Coreidae	Coreinae	Anisoscelini	*Leptoscelis*	*quadrisignatus*	8373644	3481066	8542	4150358	485.88	201	3420	937	35.05	773.0448239	201	3420
Coreidae	Coreinae	Gonocerini	*Cletomorpha*	*nyasana*	2872812	1870543	12839	5384418	419.38	185	3201	1012	37.86	528.2420949	201	2680
Coreidae	Coreinae	Gonocerini	*Cletus*	*binotulatus*	7923198	4837846	31863	13816082	433.61	179	6361	1238	46.32	700.5161551	201	2914
Coreidae	Coreinae	Gonocerini	*Cletus*	sp.	6334316	3672259	24664	10477547	424.81	187	3685	1358	50.80	672.6634757	201	2763
Coreidae	Coreinae	Homoeocerini	*Homoeocerus*	*angulatus*	4534196	2665879	15888	7377455	464.34	184	3888	1325	49.57	737.749434	202	3069
Coreidae	Coreinae	Homoeocerini	*Homoeocerus*	*marginellus*	7809332	3508693	9155	4848962	529.65	177	4789	1010	37.79	898.8217822	202	3853
Coreidae	Coreinae	Homoeocerini	*Prismatocerus*	*auriculatus*	5330848	2826069	16421	7438508	452.99	180	3630	1267	47.40	693.8145225	201	3114
Coreidae	Coreinae	Hypselonotini	*Anasa*	*andresii*	3134698	1627650	6367	3163545	496.87	194	3110	814	30.45	743.002457	201	3056
Coreidae	Coreinae	Hypselonotini	*Anasa*	*scorbutica*	8552484	3240607	9145	4354582	476.17	194	4210	902	33.74	752.304878	201	4210
Coreidae	Coreinae	Hypselonotini	*Hypselonotus*	*lineatus*	5723868	2286214	4409	1990205	451.40	191	5170	561	20.99	528.2869875	201	2892
Coreidae	Coreinae	Hypselonotini	*Hypselonotus*	*punctiventris*	2173082	1223039	9150	4291551	469.02	201	4267	1063	39.77	608.8024459	203	2660
Coreidae	Coreinae	Mictini	*Anoplocnemis*	*curvipes*	8379830	3695282	10417	5077522	487.43	189	5769	880	32.92	801.7534091	201	2938
Coreidae	Coreinae	Mictini	*Anoplocnemis*	*phasianus*	16602494	8207329	37865	15132165	399.63	183	4017	1441	53.91	739.1075642	201	2978
Coreidae	Coreinae	Mictini	*Mictis*	*longicornis*	1646384	995761	6507	2411749	370.64	192	2774	1288	48.19	438.996118	201	2774
Coreidae	Coreinae	Mictini	*Mictis*	*profana*	8903460	3767996	9157	4613789	503.85	183	4162	1000	37.41	765.077	203	3191
Coreidae	Coreinae	Phyllomorphini	*Pephricus*	*paradoxus*	11113622	7678651	37585	15828252	421.13	199	3592	1379	51.59	769.5358956	202	3081
Coreidae	Coreinae	Spartocerini	*Spartocera*	*batatas*	6613952	2946003	9815	5034034	512.89	188	5472	932	34.87	915.3927039	202	5472
Lygaeidae	Lygaeinae		*Oncopeltus*	*cingulifer*	19949254	6615116	19336	8224437	425.34	189	2710	1041	38.95	847.4082613	204	2226

Table S1. Summary data for sequence reads, contigs, and ultraconserved element loci generated in this study.

	Estimated Coefficent	Standard Error	t	p
Intercept	8.5790702	8.81583	0.973144	0.334742
Latitude	0.5033034	0.2550053	1.973698	0.053448
Body Size	3.3586916	1.143077	2.93829	0.004815
Enlarged Leg	−23.1199948	6.5951357	−3.50561	0.000915

Table S2. Best model for the median female data.

	Estimated Coefficent	Standard Error	t	p
Intercept	7.24364	7.54845	0.95962	0.341447
Latitude	0.63452	0.22876	2.77373	0.007553
Body Size	2.94169	0.94109	3.12584	0.002831
Enlarged Leg	-16.80564	5.12578	-3.27865	0.001812

Table S3. Best model for the mean female data.

	Estimated Coefficent	Standard Error	t	p
Intercept	10.776588	8.73097	1.234295	0.222438
Latitude	0.452489	0.257162	1.759548	0.084149
Body Size	2.87045	1.230257	2.333212	0.023391

Table S4. Best models for the median male data

	Estimated Coefficent	Standard Error	t	p
Intercept	10.143178	15.851696	0.63988	0.525115
Latitude	1.483707	0.553177	2.682154	0.009833
Body Size	2.698446	3.145863	0.857776	0.395029
Enlarged Leg	-31.93852	12.66494	-2.521806	0.014841
Latitude:Body Size	-0.175682	0.097263	-1.80626	0.07678
Enlarged Leg:Body Size	5.551336	2.429915	2.28458	0.026531

Table S5. Best model for the mean male data.

	Estimated Coefficent	Standard Error	t	p
Intercept	-6.470711	14.53343	-0.445229	0.658038
Latitude	1.37477	0.536069	2.564539	0.013318
Body Size	7.520353	2.763235	2.721576	0.008867
Enlarged Leg	-10.397952	5.961791	-1.744099	0.087167
Latitude:Body Size	-0.16018	0.094221	-1.700055	0.095212

Table S6. Best model for the mean male data, minus *Pephricus paradoxus.*

	Estimated Coefficent	Standard Error	t	p
Intercept	−14.0899983	16.3481031	−0.861874	0.392792
Latitude	1.4204894	0.6012626	2.3625109	0.022005
Body Size	9.1506415	3.0987825	2.9529796	0.004749
Enlarged Leg	−10.370064	6.8008545	−1.524818	0.133482
Latitude:Body Size	−0.1838082	0.1052683	−1.746093	0.086816

Table S7. Best model for the median male data, minus *Pephricus paradoxus.*

LITERATURE CITED

Johnson, K. P., C. H. Dietrich, F. Friedrich, R. G. Beutel, B. Wipfler, R. S. Peters, J. M. Allen, M. Petersen, A. Donath, K. K. O. Walden, A. M. Kozlov, L. Podsiadlowski, C. Mayer, K. Meusemann, A. Vasilikopoulos, R. M. Waterhouse, S. L. Cameron, C. Weirauch, D. R. Swanson, D. M. Percy, and K. Yoshizawa. 2018. Phylogenomics and the evolution of hemipteroid insects. Proc. Natl. Acad. Sci. 115:12775–12780.

Liu, Y., H. Li, F. Song, Y. Zhao, J. Wilson, and W. Cai. 2019. Higher-level phylogeny and evolutionary history of Pentatomomorpha (Hemiptera: Heteroptera) inferred from mitochondrial genome sequences. Syst. Entomol.

Li, H., J. M. Leavengood, E. G. Chapman, D. Burkhardt, F. Song, P. Jiang, J. Liu, X. Zhou, and W. Cai. 2017. Mitochondrial phylogenomics of Hemiptera reveals adaptive innovations driving the diversification of true bugs. Proc. Biol. Sci. 284.

Wang, Y., Y. Cui, D. Rédei, P. Baňař, Q. Xie, P. Štys, J. Damgaard, P. Chen, W. Yi, Y. Wang, K. Dang, C. Li, and W. Bu. 2016. Phylogenetic divergences of the true bugs (Insecta: Hemiptera: Heteroptera), with emphasis on the aquatic lineages: the last piece of the aquatic insect jigsaw originated in the Late Permian/Early Triassic. Cladistics 32:390–405.
